# Impact of sex and comorbid diabetes on hospitalization outcomes in acute pancreatitis: A large United States population-based study

**DOI:** 10.3934/publichealth.2023009

**Published:** 2023-02-23

**Authors:** Simcha Weissman, Stephen J. Pandol, Umar Ghaffar, Melody Boafo, Chukwuemeka E. Ogbu, Tamer Zahdeh, Mohammed Ashary, Vignesh Krishnan Nagesh, Anushka Kigga, Ayrton Bangolo, Aditi Bhargava

**Affiliations:** 1 Department of Medicine, Hackensack Meridian Health Palisades Medical Center, North Bergen, NJ, USA; 2 Division of Gastroenterology, Cedars-Sinai Medical Center, Los Angeles, CA, USA; 3 Center for Reproductive Sciences, Department of Obstetrics and Gynecology, University of California San Francisco, San Francisco, CA, USA

**Keywords:** acute pancreatitis, diabetes, hospitalization outcomes, race, renal failure, sex differences

## Abstract

**Backgrounds:**

Data on the association between comorbid diabetes mellitus (DM) and acute pancreatitis (AP) remains limited. Utilizing a large, nationwide database, we aimed to examine the impact of comorbid diabetes mellitus on patients admitted for acute pancreatitis.

**Methods:**

This was a retrospective case-control study of adult patients with AP utilizing the National Inpatient Sample from 2015–2018, using ICD–10 codes. Hospitalization outcomes of patients admitted for AP with comorbid DM were compared to those without comorbid DM at the time of admission. The primary outcome was a mortality difference between the cohorts. Multivariable-adjusted cox proportional hazards model analysis was performed. Data was analyzed as both sex aggregated, and sex segregated.

**Results:**

940,789 adult patients with AP were included, of which 256,330 (27.3%) had comorbid DM. Comorbid DM was associated with a 31% increased risk of inpatient mortality (aOR: 1.31; p = 0.004), a 53% increased risk of developing sepsis (aOR: 1.53; p = 0.002), increased hospital length of stay (LOS) (4.5 days vs. 3.7 days; p < 0.001), and hospital costs ($9934 vs. $8486; p < 0.001). Whites admitted for AP with comorbid DM were at a 49% increased risk of mortality as compared to Hispanics (aOR: 1.49; p < 0.0001). Different comorbidities had sex-specific risks; men admitted for AP with comorbid DM were at a 28% increased risk of mortality (aOR: 1.28; p < 0.0001) as compared to women. Men with comorbid DM plus obesity or hypertension were also at increased risk of mortality as compared to women, whereas women with comorbid DM plus renal failure were at greater risk of mortality as compared to men.

**Conclusions:**

Comorbid DM appears to be a risk factor for adverse hospitalization outcomes in patients admitted for AP with male sex and race as additional risk factors. Future prospective studies are warranted to confirm these findings to better risk stratify this patient population.

## 1. Introduction

Acute pancreatitis is a common indication for inpatient hospital care in the United States with an annual incidence of 13–45 cases per 100,000 persons [1]–[3]. The focus of the management of acute pancreatitis has been on rapid diagnosis; the determination of the severity of the disease for determining the level of care required; and consideration of causative factors for addressing during acute management and planning prevention of recurrent attacks [1]. Sex differences exist in the etiology of pancreatitis; alcohol and tobacco predominate in men, whereas idiopathic and obstructive etiologies predominate in women [4].

In addition to severity determination, there are several clinical factors that increase the risk of complications or death with an episode of acute pancreatitis. These factors include advanced age (≥60 years), obesity and a history of heavy alcohol use [5]. Numerous and severe coexisting conditions as measured by an increased Charlson comorbidity index (a score of ≥2) are associated with increased morbidity and length of stay with an episode of acute pancreatitis [5]–[7]. Although the Charlson comorbidity index includes the presence of diabetes and diabetes with complications, we are not aware of any studies that show the specific effect of comorbid diabetes on the course of acute pancreatitis in men and women.

The prevalence of diabetes has increased significantly in the United States over the past three decades [8] suggesting that greater numbers of patients presenting with acute pancreatitis have comorbid diabetes. This trend prompted us to determine the effect of comorbid diabetes on the course of acute pancreatitis. Furthermore, because the prevalence of diabetes is greater in men than women [9], we wanted to do determine if there is a sex difference of the impact of comorbid diabetes on outcomes in acute pancreatitis.

To address these questions, we utilized a large nationwide database to investigate the impact of comorbid diabetes mellitus (DM) in men and women admitted for management of an episode of acute pancreatitis (AP).

## 2. Materials and methods

### 2.1. Data source

In this retrospective case-control study, we utilized the National Inpatient Sample (NIS) database from 2015–2018. The NIS is the largest publicly available database of inpatient stays derived from billing data based upon discharge abstracts. The NIS database contains data from over 4500 hospitals in 48 US states and is thus considered to be nationally representative. It contains de-identified clinical and nonclinical elements at both the patient and hospital level and can be queried based upon International Classification of Diseases, tenth Revision, Clinical Modification (ICD–10 CM) coding terms.

### 2.2. Ethical consideration

This study did not require institutional review board approval as it uses publicly available de-identified data.

### 2.3. Study population and inclusion criteria

Patients hospitalized for a primary diagnosis of AP (based upon the ICD–10 code K85) between 2015–2018 were selected from the general population. All patients under the age of 18, admissions that were elective, or patients with incomplete information on sex, age, or demographics, were excluded. Thereafter, those patients admitted for AP were stratified based upon the presence or absence of comorbid DM at the time of hospitalization. Patient and hospital-level characteristics were compared between these two groups (those with vs. without comorbid DM). Additionally, the impact of comorbid DM upon hospitalization outcomes were assessed. Finally, in the cohort of patients admitted for AP who also had comorbid DM, we examined the effect age, sex, demographics, and other common comorbidities had on hospital outcomes.

### 2.4. Study variables

Variables included patient age (>65 vs. <65); sex (Women vs. Men); race (Black, Hispanic, Native American, Asian-Pacific Islander vs. white); the presence of obesity; the presence of hypertension; and the presence of renal impairment. All variables were assessed for their impact on inpatient mortality in the cohort of patients hospitalized for AP who also had comorbid DM. Burden of comorbidities was assessed using the Elixhauser comorbidity indices.

### 2.5. Primary and secondary outcomes

The primary outcome was the inpatient mortality and sex differences in patients admitted for AP with vs. without comorbid DM. Secondary outcomes included the difference in (a) mean hospitalization LOS, (b) mean hospitalization cost, (c) risk of sepsis (based upon the ICD–10 codes A40 and A41), and (d) discharge disposition between these two cohorts.

### 2.6. Statistical analysis

Statistical analyses were performed using SAS 9.4 (SAS Institute Inc, Cary, North Carolina). Weighting of patient-level observations was implemented. Univariate analysis was initially performed to calculate an unadjusted odds ratio and determine confounders significantly associated with the outcomes. Multivariable-adjusted cox proportional hazards model analysis was used to adjust for potential confounders. A multivariable-adjusted cox proportional hazards regression model was then built by including all confounders that were found to be significant by univariate analysis, to calculate an adjusted odds ratio. Sex was then also used as an independent variable to perform analyses related to the mortality difference amongst cohorts (i.e., those with vs. without the comorbidities of interest). Proportions were compared using chi-square test for categorical variables, and Student's t-test for continuous variables. All p-values were two-sided, with 0.05 as the threshold for statistical significance. 30 comorbidities were taken into account among which: Congestive heart failure, Cardiac arrythmias, Valvular disease, Pulmonary circulation disorders, peripheral vascular disorders, Hypertension, paralysis, neurodegenerative disorders, uncomplicated diabetes, complicated diabetes, hypothyroidism, renal failure, liver disease, peptic ulcer disease excluding bleeding, AIDS/HIV, lymphoma, metastatic cancer, solid tumor without metastasis, rheumatoid arthritis/collagen vascular diseases, coagulopathy, obesity, weight loss, fluid and electrolyte disorders, blood loss anemia, deficiency anemia, alcohol abuse, drug abuse, Psychoses, and depression.

## 3. Results

### 3.1. Patient demographics and hospital characteristics

940,789 adult patients with a diagnosis of AP were included in the study. Of these, 256,330 (27.3%) had comorbid DM and 684,460 (72.7%) did not. The cohort of patients whom had comorbid DM were significantly older (mean age of 55.6 years vs. 49.5; p < 0.0001), more likely to be men (56.7% vs. 51.9%; p < 0.001), obese (27.2% vs. 13.4%; p < 0.0001), have hypertension (77.4% vs. 48.1%; p < 0.001), and have renal failure (16% vs. 5.6%; p < 0.001); and less likely to be white (54.8% vs. 64.5%; p < 0.001) compared to the cohort of patients whom did not have DM upon hospitalization for AP. Additional patient and hospital characteristics for both cohorts are presented in Table 1. A total of 37 different comorbidities were reported in this patient population but only 8 of those comorbidities were associated significantly with DM in AP (Table 1).

### 3.2. Mortality

The primary outcome, all-cause inpatient mortality in the cohort of patients with comorbid DM, who were admitted for AP, was observed in 0.67% of admissions vs. 0.46% in the cohort who did not have comorbid DM. In an unadjusted analysis comorbid DM was associated with a 58% increased risk of inpatient mortality (OR: 1.58; 95% CI: 1.71–1.98; p = 0.005). Upon multivariable analysis, comorbid DM was associated with a 31% increased risk of inpatient mortality (aOR: 1.31; 95% CI: 1.84–1.97; p = 0.004) (Table 2).

Men admitted for AP who had comorbid DM were at a 28% increased risk of mortality (0.71% vs 0.61%, (aOR: 1.28; 95% CI: 1.05–1.44; p < 0.0001) as compared to women. Patients >65 years of age, admitted for AP who had comorbid DM were at a 219% increased risk of mortality (1.43% vs. 0.36%, aOR: 3.19; 95% CI: 2.79–3.67; p < 0.0001) as compared to those <65 years of age. Whites admitted for AP whom had comorbid DM were at a 49%, 21%, and 7% increased risk of mortality as compared to Hispanics (0.80% vs. 0.30%, (aOR: 1.49; 95% CI: 1.42–1.62; p < 0.0001), Blacks (0.80% vs. 0.60%, aOR: 1.21; 95% CI: 1.11–1.68; p < 0.0001), and AP Islanders (0.80% vs. 0.74%, aOR: 1.07; 95% CI: 1.01–1.18; p < 0.0001), respectively (Table 2).

**Table 1.** Baseline patient and hospital characteristics of the study population.

NIS 2015–2018			
Baseline characteristics N = 940,789	Acute pancreatitis with DM N = 256,330 (27.3%)	Acute pancreatitis without DM N = 684,460 (72.7%)	P-value
Age			<0.0001
Mean years (Mean ± SD)	55.6 ± 15.3	49.5 ± 17.7	
Sex			<0.0001
Men	56.7%	51.9%	
Women	43.3%	48%	
Age groups			<0.0001
<18	0.4%	2.3%	
18–34	8.6%	19.5%	
35–49	25.8%	29.1%	
50–64	36.5%	28.7%	
65–79	22.1%	14.2%	
≥80	6.6%	6.1%	
Race			<0.0001
White	54.8%	64.5%	
Black	19.4%	15.2%	
Hispanic	16.2%	12.9%	
Ap Islander	9.2%	7.2%	
Other	0.04%	0.02%	
Insurance type			<0.0001
Medicare	39.9%	26.5%	
Medicaid	21.3%	25.9%	
Private	28.7%	33.5%	
Other	10.1%	14.1%	
Elixhauser Comorbidities			
Congestive heart failure	9.9%	4.1%	<0.0001
Valvular disease	2.5%	1.9%	<0.0001
Peripheral vascular disease	3.9%	2.8%	<0.0001
Hypertension	77.4%	48.1%	<0.0001
Renal failure	16%	5.6%	<0.0001
Liver disease	19%	16.3%	<0.0001
Rheumatoid arthritis/collagen			<0.0001
Vascular disease	2.5%	2.3%	
Obesity	27.2%	13.4%	<0.0001
Hospital ownership/control			<0.0001
Rural	12.3%	11.7%	
Urban nonteaching	26.6%	26.5%	
Urban teaching	61.1%	61.7%	
Income quartile by zip code			<0.0001
0–25^th^	36.1%	31.2%	
26–50^th^	26.9%	26.7%	
51–75^th^	22.2%	23.8%	
76–100^th^	14.8%	18.2%	
Geographic region			<0.0001
Northeast	15.7%	17.1%	
Midwest	22.1%	22.5%	
South	43.1%	40%	
West	18.9%	20.4%	
Hospital Bed size			<0.0001
Small	23%	24%	
Medium	30.5%	30.6%	
Large	46.4%	45.4%	

**Table 2.** Multivariable analyses of factors affecting mortality in Diabetic patients admitted for Acute pancreatitis.

Characteristics	Mortality in patients with Acute Pancreatitis	
	Percentage (%)	95 % CI
		*P* = 0.004
Patients with DM	0.67	aOR: 1.31; (95% CI, 1.84–1.97)
Patients without DM	0.47	
Sex		*P <* 0.0001
Men with DM	0.71	aOR: 1.28; (95% CI, 1.05–1.44)
Women with DM	0.61	
Age		*P* < 0.0001
≥65 with DM	70.9	aOR: 3.19; (95% CI, 2.79–3.67)
<65 with DM	29.1	
Ethnicities comparisons among DM patients
Whites vs. Hispanics	0.80 vs. 0.30	aOR: 1.49; (95% CI: 1.42–1.62) *p* < 0.0001
Whites vs. Blacks	0.80 vs. 0.60	aOR: 1.21; (95% CI: 1.11–1.68) *p* < 0.0001
Whites vs. Pacific Islanders	0.80 vs. 0.74	aOR: 1.07; (95% CI: 1.01–1.18) *p* < 0.0001
Associated Comorbidities		
DM and obesity	0.72	aOR: 1.17; (95% CI: 1.15–1.19) *p* < 0.0001
DM and Hypertension	0.77	aOR: 1.19; (95% CI: 1.32–1.45) *p* < 0.0001
DM and renal failure	0.93	aOR: 1.31; (95% CI: 1.25–1.63) *p* < 0.0001

**Figure 1.**
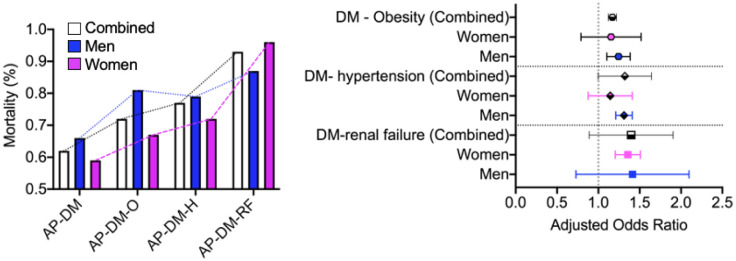
Sex aggregated and segregated data pertaining to mortality associated with comorbid Diabetes mellitus (DM) plus a secondary comorbidity vs. those with only comorbid DM.

Compared to those with only comorbid DM who were admitted for AP, patients were at a 17%, 19%, and 31% increased risk of mortality if they also had comorbid obesity (0.62% vs. 0.72%, aOR: 1.17; 95% CI: 1.15–1.19; p < 0.0001), hypertension (0.62% vs. 0.77%, aOR: 1.19; 95% CI: 1.32–1.45; p < 0.0001), or renal failure (0.62% vs. 0.93%, aOR: 1.31; 95% CI: 1.25–1.63; p < 0.0001), respectively. Data segregated by sex showed that men admitted for AP were at a greater risk of mortality if they also had comorbid DM, DM plus obesity, and DM plus hypertension than women admitted for AP with matched comorbidities (Figure 1). In contrast, women admitted for AP were at a greater risk of mortality if they also had comorbid DM plus renal failure compared to men (Figure 1).

**Figure 2.**
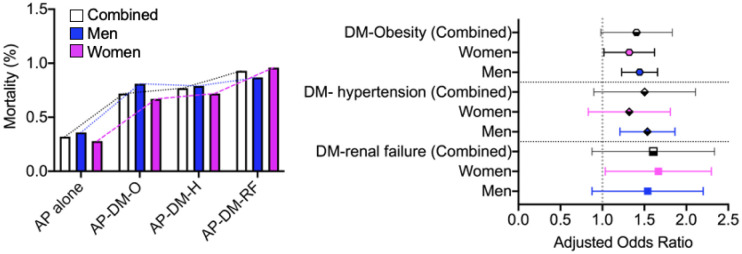
Sex aggregated and segregated data pertaining to mortality associated with Comorbid Diabetes mellitus (DM) plus comorbid obesity, hypertension, or renal failure vs. those without any of these comorbidities.

Compared to those without comorbid DM, patients admitted for AP whom had comorbid DM were at a 38%, 41%, and 66% increased risk of mortality if they also had comorbid obesity (32% vs. 0.72%, aOR: 1.38; 95% CI: 1.25–1.59; p < 0.0001), hypertension (32% vs. 0.77%, aOR: 1.41; 95% CI: 1.32–1.78; p < 0.0001), or renal failure (32% vs. 0.93%, aOR: 1.66; 95% CI: 1.29–1.87; p < 0.0001), respectively. Data segregated by sex showed men and women have different risks for mortality (Figure 2).

**Figure 3.**
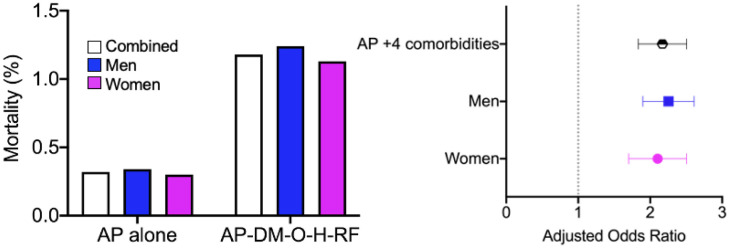
Sex aggregated and segregated data pertaining to mortality associated with Comorbid Diabetes mellitus (DM) plus obesity, hypertension, and renal failure compared to those without any of these comorbidities.

Compared to those without any of the aforementioned comorbidities, patients admitted for AP who had comorbid DM, obesity, hypertension, and renal failure together were at a 116% increased risk of mortality (0.32% vs. 1.18%, aOR: 2.16; 95% CI: 2.04–2.31; p = 0.007). Data segregated by sex revealed differences in risks for different comorbidities (Figure 3).

### 3.3. Sex differences in other comorbidities for AP outcomes

Next, we analyzed if there were sex differences in AP outcomes with or without other comorbidities. Men compared to women with comorbid DM plus obesity or hypertension vs. none of these comorbidities had an aOR 1.15, 95% CI: 0.77–1.13, p = 0.18 and an aOR 1.21, 95% CI: 0.11–1.41, p = 0.004, respectively. Men compared to women with comorbid DM plus obesity or hypertension vs. only comorbid DM had an aOR 1.19, 95% CI: 1.13–1.47, p < 0.001 and an aOR 1.26, 95% CI: 0.1.17–1.35, p = 0.002, respectively. Women compared to men with comorbid DM plus renal failure vs. none of these comorbidities had an aOR 1.09, 95% CI: 0.97–1.22, p = 0.27 (not significant). In contrast, women compared to men with comorbid DM plus renal failure vs. only comorbid DM had an aOR 1.08, 95% CI: 1.02–1.18, p = 0.01.

### 3.4. Sepsis

Sepsis during hospitalization in the cohort of patients with comorbid DM, who were admitted for AP, was observed in 1.86% of admissions vs. 1.13% in the cohort who did not have comorbid DM. In an unadjusted analysis comorbid DM was associated with a 132% increased risk of developing sepsis (OR: 2.32; 95% CI: 2.11–2.83; p = 0.005). Upon multivariable analysis, comorbid DM was associated with a 53% increased risk of developing sepsis (aOR: 1.53; 95% CI: 1.09–1.55; p = 0.002).

### 3.5. Healthcare utilization outcomes

The cohort of patients admitted for AP who had comorbid DM, had significantly increased hospital LOS (4.5 days vs. 3.7 days; p < 0.001, hospital costs ($9934 vs. $8486; p < 0.001), and decreased odds of discharge to home (81.7% vs. 87.4%, p < 0.001) as compared to the cohort who did not have comorbid DM. Upon multivariable analysis, comorbid DM was associated with 18% lower odds of being discharged to home (aOR: 0.82; 95% CI: 0.73–0.94; p = 0.002).

## 4. Discussion

This is a first large retrospective case-control study of adult patients with AP to report that comorbid diabetes mellitus is associated with a 31% increased risk of inpatient mortality, a 53% increased risk of developing sepsis, increased hospital length of stay, and hospital costs. A total of 37 different comorbidities were reported in this patient population but we found only 8 of those comorbidities were associated significantly with DM in AP. Overall, men compared with women with AP and comorbid DM, obesity and/or hypertension had worse outcomes and increased mortality. In contrast, women compared with men with AP and comorbid renal failure had worse outcomes and increased mortality. Differences in ethnicity and race were also noted.

While several risk factors are shared between men and women for AP, our data suggests that diabetes, obesity, and hypertension render men more susceptible to worse outcomes for AP, whereas renal failure makes women more susceptible to worse outcomes for AP. In men worldwide, factors such as smoking, alcohol, abdominal obesity, and diabetes account for the overall increased risk of developing AP. Women on the other hand, show a greater risk of biliary pancreatitis and post-endoscopic retrograde cholangiopancreatography pancreatitis [10],[11]. Sex differences in AP may arise due to anatomical differences in pancreas size between men and women.

Another retrospective study revealed that women compared to men with AP were less likely to die, had lower incidence of sepsis, shock, acute kidney injury, and pancreatic drainage than men with AP [12]. While ICU admissions incidence were lower women with AP than men, the mean length of stay and hospital charges and cost did not differ by sex [12].

Increased risk of severe acute pancreatitis is associated with intra-abdominal, omental fat and intra-pancreatic fat distribution [13],[14]. While men compared to women have a greater propensity for developing intra-abdominal fat, other studies report that risk for severe AP is similar in men and women with similar intra-abdominal fat content [15]. Our data suggests that men compared to women with comorbid obesity have worse AP outcomes and have a higher mortality rate. Higher intra-pancreatic fat mass increases the risk for severe acute pancreatitis, [13] but sex differences have not been studied in the context of intra-abdominal versus intra-pancreatic fat mass distributions; mechanisms remain largely unexplored [14].

## 5. Conclusions

In conclusion, we report that comorbid diabetes, obesity, hypertension, and renal failure increases the risk of mortality and worse outcomes for patients with acute pancreatitis; only 8 out of 30 comorbid conditions worsen AP outcomes. Comorbid diabetes, obesity, and hypertension have worse outcomes for men, whereas comorbid renal failure has worse outcomes for women. When evaluating treatment regimens, these comorbid conditions and outcomes should be taken into account.
